# Genome-Wide Identification and Expression Analysis of the *SPL* Gene Family in *Phalaenopsis equestris*

**DOI:** 10.3390/plants14193090

**Published:** 2025-10-07

**Authors:** Xule Zhang, Lei Feng, Qingdi Hu, Yaping Hu, Xiaohua Ma, Jian Zheng

**Affiliations:** Key Laboratory of Plant Innovation and Utilization, Zhejiang Institute of Subtropical Crops, Wenzhou 325005, China; zhangxl@zaas.ac.cn (X.Z.); chinafenglei@163.com (L.F.); huqd@zaas.ac.cn (Q.H.); huyp@zaas.ac.cn (Y.H.)

**Keywords:** PeqSPLs, orchid, phylogenetic analysis, conserved domain, functional differentiation

## Abstract

The SQUAMOSA promoter-binding protein-like (SPL/SBP) family plays crucial roles in multiple developmental processes. *Phalaenopsis equestris* is a key ornamental and breeding species known for producing abundant colorful flowers on a single inflorescence. The *SPL* gene family in this species remains largely uncharacterized. In this study, 15 *SPL* genes were identified, all encoding proteins that are bioinformatically predicted to be nuclear-localized, hydrophilic, and unstable, with conserved SBP domains. Phylogenetic and collinearity analyses revealed a closer evolutionary relationship with rice *SPLs* than Arabidopsis *SPLs*. Conserved motif and gene structure analyses showed that subfamily II members possess more motifs and introns, implying functional complexity. Five PeqSPLs contained transmembrane domains, suggesting potential dual nuclear/cytoplasmic roles. Promoter analysis revealed abundant *cis*-elements responsive to light, stress, and phytohormones. Expression profiling across tissues showed that *PeqSPL2*, *PeqSPL3*, and *PeqSPL5* exhibited broad expression and *PeqSPL10* exhibited predominantly high expression in flowers, indicating possible roles in normal growth and floral development. This study provides a foundation for further functional exploration of *PeqSPL* genes in *P. equestris*.

## 1. Introduction

SQUAMOSA promoter-binding protein-like (SPL) transcription factors constitute a plant-specific family characterized by the conserved SBP DNA-binding domain [1,2]. This domain features dual zinc fingers (Zn-1: C_3_H, Zn-2: C_2_HC) and a bipartite nuclear localization signal (NLS), enabling DNA recognition and nuclear targeting [3]. First identified in *Antirrhinum majus* for regulating floral meristem gene *SQUAMOSA*, *SPLs* are evolutionarily conserved from green algae (Chlamydomonas CRR1) to angiosperms [4,5]. The SPL transcription factor family is exclusive to plants and plays crucial roles in multiple developmental processes [5]. Additionally, SPLs are involved in plant responses to both biotic and abiotic stresses [6].

In *Arabidopsis thaliana*, 16 *SPL* genes have been identified and classified into eight groups, with many members functioning in vegetative phase transitions, flowering induction, floral organ development, root formation, trichome initiation, and responses to abiotic stresses such as cold, salt, drought, and heat [7,8,9,10,11]. Notably, many *SPL* genes are regulated by miR156/157, forming a crucial regulatory module that integrates developmental cues with stress and hormonal signals [12]. *AtSPL3*, *AtSPL4*, and *AtSPL5* are involved in promoting floral meristem development [13]. *AtSPL14* contributes to fumonisin B1 resistance, while *AtSPL8* influences gibberellic acid biosynthesis and regulates reproductive development [10,11,14]. In rice, 19 *OsSPL* gene and 12 OsmiR156 precursors were identified [15]. *OsSPL10* enhances drought tolerance by regulating *OsNAC2* and ROS production in rice [16]. *OsSPL14* controls plant architecture by reducing tiller number, increasing grain weight, and enhancing disease resistance [17,18]. *OsSPL16* promotes grain filling and improves both yield and quality [19]. *OsSPL3* has been shown to enhance cold tolerance [20]. *OsSPL2*, *OsSPL4*, *OsSPL16*, and *OsSPL17* regulate temperature-sensitive male fertility via the miR156-*SPL* module [21]. In maize, 30 *ZmSPL* genes were identified, and 18 of them were predicted as putative miR156 targets [22]. *ZmSPL10*, *ZmSPL14*, and *ZmSPL26* redundantly regulate leaf trichome formation in maize. In their triple mutant, the leaves display a glabrous phenotype, and hair cells are transformed into stomata [23]. *ZmSPL13* and *ZmSPL29* were identified as key regulators of vegetative phase change and flowering time in maize, linking the miR156-*SPL* and miR172-*Gl15* modules and offering potential targets for crop breeding [22]. Fifty-six *PtSPL* genes were identified in *Populus tomentosa*, and their expression patterns suggest roles in tissue and reproductive development [5]. *SPL* also plays important roles in gymnosperms; thirteen *SPL* genes were identified in *Ginkgo biloba*, and expression analyses suggest their involvement in flavonoid biosynthesis and water stress responses [3]. This indicates that *SPL* family genes play crucial roles in plant growth and development, morphogenesis, reproductive processes, and stress responses across both gymnosperms and angiosperms.

Orchidaceae is a large angiosperm family known for diverse floral morphology, specialized pollination, drought adaptation, and complex mycorrhizal associations. With terrestrial, epiphytic, and saprophytic forms, orchids are ideal for studying biodiversity and evolution [24]. A previous study identified 16 *SPL* genes in *Cymbidium goeringii*, 17 in *Dendrobium chrysotoxum*, and 10 in *Gastrodia elata,* and analyzed their structures, expression patterns, and regulatory roles in floral development [25]. Most SPLs contained conserved SBP domains and light-responsive cis-elements, with *DchSPL9* and *GelSPL2* showing significant expression changes during flowering. Phalaenopsis species are widely favored as ornamental plants due to their graceful appearance and long-lasting blooms, making them economically valuable in the floral industry. *Phalaenopsis equestris* serves as a key breeding parent for its ability to produce numerous colorful flowers on a single inflorescence. *P. equestris* underwent whole-genome sequencing as early as 2015, but the functions and characteristics of its *SPL* genes remain largely unexplored, to date.

In this study, phylogenetic analysis, conserved protein domain analysis, gene structure analysis, *cis*-element analysis, and expression pattern analysis of the *SPL* gene family in *P. equestris* were carried out to illustrate the conservation and variation in *PeqSPLs*.

## 2. Results

### 2.1. Identification of PeqSPL Family Members

A Hidden Markov Model (HMM) was constructed from known SPL protein family sequences of *A. thaliana* and rice using HMMER 3.0. This model was subsequently employed to interrogate the full complement of coding protein sequences in *P. equestris* to identify putative SPL family homologs. In parallel, all *P. equestris* protein sequences were aligned against reference SPL sequences via BLAST analysis. Through this dual screening strategy employing HMM-based profiling and sequence similarity alignment, 15 genes encoding complete SPL domains were ultimately identified and validated in *P. equestris*. The *PeqSPL* genes were named according to the gene ID order (Table 1). The peptides length of PeqSPLs ranges from 181 aa to 1106 aa, and the MW ranges from 19.91 kDa to 121.98 kDa. The pI of most PeqSPLs was higher than 7, and nine PeqSPLs’ pI were higher than 8, which means more than half of the 15 PeqSPLs are basic proteins (pI higher than 8.00). An II of protein less than 40 predicts that it is a stable protein, whereas values higher than 40 denote a potentially unstable protein [25]. The II of all PeqSPL proteins were higher than 40, indicating they all were unstable proteins. In addition, the 15 PeqSPLs were all hydrophilic proteins because their GRAVY value were negative. The subcellular localization of all these PeqSPL proteins was predicted to be in the nucleus, which is consistent with their transcription factor function. This information tells us that although these PeqSPLs have large differences in protein size, their physicochemical properties still have similarities, indicating that PeqSPLs may be conservative. In addition, Table 1 also lists the closest homologs of each PeqSPL, of which functions have been extensively studied in model plants such as Arabidopsis and rice, providing important references for functional studies of PeqSPLs.

### 2.2. Phylogenetic Relationships and Evolutionary Divergence of PeqSPL Genes

**Figure 1:**
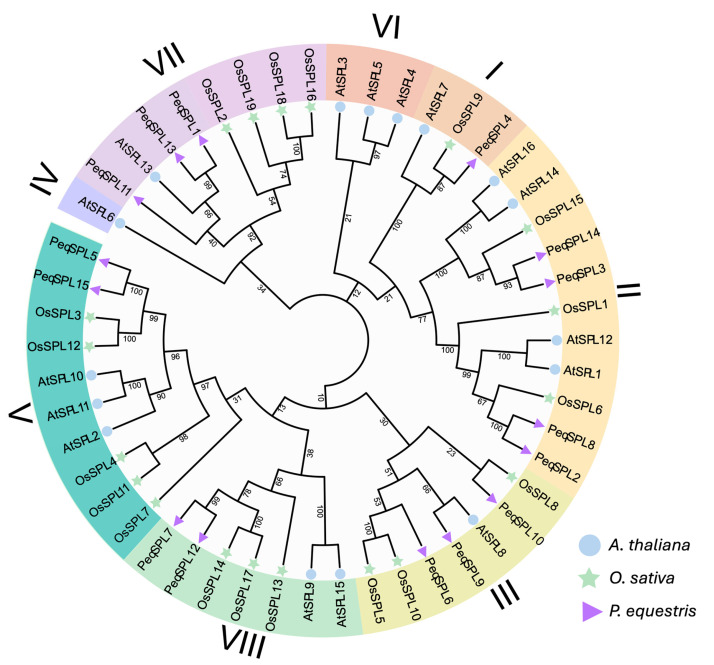
A phylogenetic tree of the *SPL* gene family from *P. equestris*, *O. sativa*, and *A. thaliana* constructed based on the full-length sequences of SPL proteins. The SPL gene family is divided into eight subfamilies (I–VIII). The PeqSPL protein sequences are shown in Table S1.

To investigate the evolutionary relationship of the *PeqSPL* genes, an NJ phylogenetic tree was constructed using the full-length protein sequences of 15 PeqSPLs, 16 AtSPL, and 19 OsSPL (Figure 1). These SPLs were divided into eight subfamilies, and the IV and VI subfamilies only contained the members of *AtSPL*. Phylogenetic analysis of the *SPL* gene family demonstrates that *P. equestris* exhibits closer affinity to rice (a fellow monocotyledonous plant) than to Arabidopsis (a dicotyledonous plant). Subfamily II contained the largest number of *PeqSPL* members, with four members, followed by III and VII. However, the subfamilies that contained the largest number of *OsSPL* members are V and VII, with five and four members, respectively. This indicates that during the process of evolution, the *SPL* gene families of different monocotyledonous plants have also undergone divergent expansion and contraction.

### 2.3. Protein Conservative Domain and Functional Motifs Analysis

**Figure 2:**
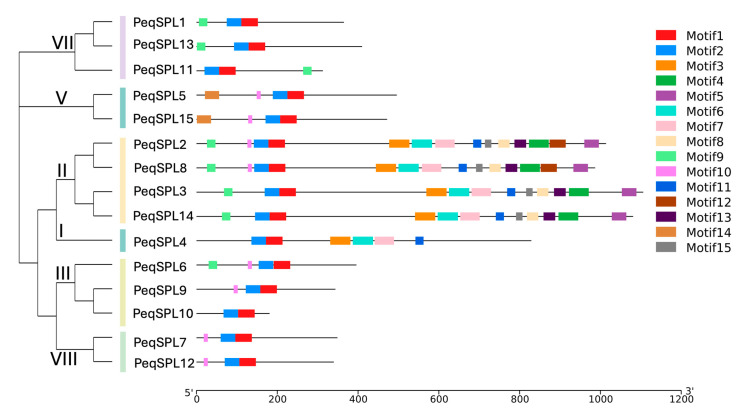
Conserved motifs in PeqSPL proteins predicted by MEME. Lines of different colors on the left represent different *SPL* subfamilies.

**Figure 3:**
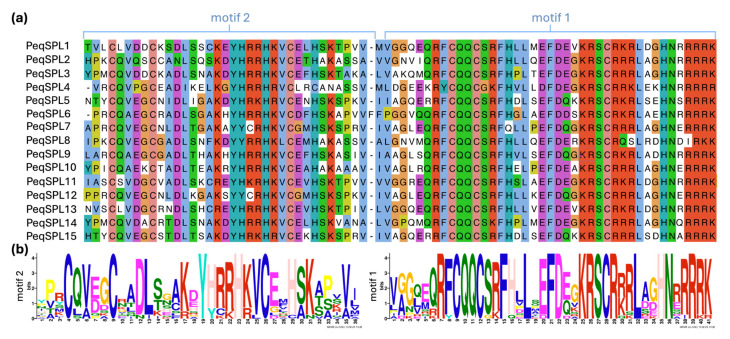
Conserved motifs in *PeqSPL* amino acid sequences. (**a**) Sequence alignment results of PeqSPL proteins. (**b**) Sequence logo of the conserved motif 1 and motif 2.

To further analyze *PeqSPLs*, we conducted bioinformatics predictions on the protein-conserved motif of 15 PeqSPL proteins and identified 15 conserved motifs, named motif 1 to motif 15 (Figure 2 and Figure S1). The distribution of these motifs reflects the phylogenetic classification of PeqSPLs, and members of the same subfamily have similar motif characteristics. There are some differences in conserved motifs between different subfamilies. Motif 1 and motif 2 are present in all PeqSPLs, indicating that these two motifs are the most conserved, and they may be related to the core function domain of SPL protein (Figure 3). Motif 9 and motif 10 were found in eight PeqSPLs, respectively, and only PeqSPL4 and PeqSPL10 did not contain either of them. Members of subfamilies I and II possess relatively long amino acid sequences (>800 aa) and share similar conserved motifs. Motif 3, motif 6, motif 7, and motif 11 are conserved in both subfamilies. Additionally, all members of subfamily II exclusively contain five motifs: motif 4, motif 5, motif 8, motif 13, and motif 15. More motifs provide the possibility for proteins to have more functions. The members of the subfamily II contain at least 12 motifs, so they may have more abundant functions than other PeqSPLs.

Additionally, subcellular localization predictions for the PeqSPL family members were performed, and the results indicated that all PeqSPL proteins are localized to the nucleus (Table S2), consistent with their function as transcription factors. Proteins containing transmembrane domains (TMDs) may function as membrane receptors, membrane-anchored proteins, or ion channels localized to cellular membranes. Prediction revealed that five PeqSPL proteins (PeqSPL2, PeqSPL3, PeqSPL4, PeqSPL8, and PeqSPL14) each possess one TMD (Table S2). Since PeqSPL4 is the sole member of subfamily I and PeqSPL2, PeqSPL3, PeqSPL8, and PeqSPL14 constitute all members of subfamily II, it can be concluded that all proteins in both subfamilies I and II contain TMD. This suggests that PeqSPL proteins in these subfamilies may share conserved functions beyond their canonical roles as nuclear transcription factors, potentially including membrane-associated activities.

### 2.4. Gene Structure Variation Among PeqSPL Family Members

**Figure 4:**
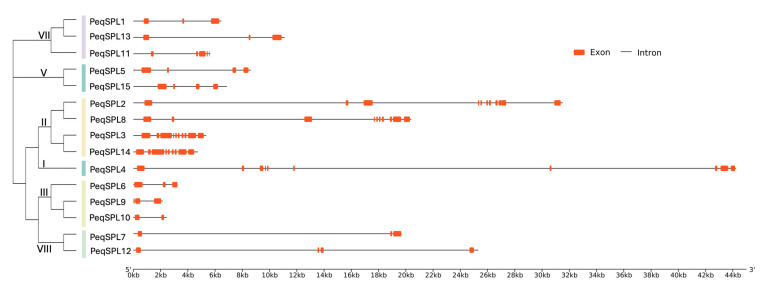
Gene structure visualization of the *PeqSPL* genes. Visualization of *PeqSPLs* gene structure. Lines of different colors on the left represent different *SPL* subfamilies.

We also analyzed the gene structure of each member of the *PeqSPLs* family. The 15 PeqSPLs genes contain different number and length of introns (Figure 4). However, the members of the unified subfamily have relatively high similarity in gene structure. The *PeqSPL* genes with the largest number of introns all belong to subfamily II. *PeqSPL2* contains 10 introns, which is the most of all *PeqSPL* genes, followed by *PeqSPL3*, *PeqSPL4*, and *PeqSPL8*, each containing nine introns. The *PeqSPL10* only contains one intron, followed by *PeqSPL6*, *PeqSPL7*, *PeqSPL9*, and *PeqSPL13*, each containing two introns, and *PeqSPL6*, *PeqSPL9*, and *PeqSPL10* belong to subfamily III. In general, most *PeqSPL* genes contain no more than four introns. Additionally, the intron of *PeqSPL4* is the longest (>18 kb), and the introns of PeqSPL3 and PeqSPL14 are the shortest. The difference in the length and arrangement of exons and introns of the *PeqSPL* genes make them more variable.

### 2.5. Collinearity and Synteny Analysis of PeqSPL Genes

**Figure 5:**
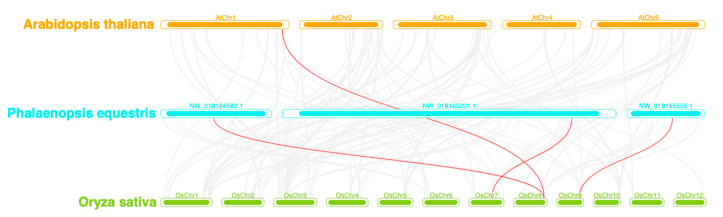
Collinearity analysis of SPL genes between *P. equestris* with *A. thaliana* and *O. sativa*. The horizontal bars represent the chromosomes of *A. thaliana* (**top**), *P. equestris* (**middle**), and *O. sativa* (**bottom**). Syntenic relationships are represented by red lines connecting corresponding chromosomes, indicating conserved gene sequence and suggesting evolutionary conservation between the species.

Intra-species collinearity analysis is presented in Figure S2. The results indicate no detectable collinear pairs (0 collinear relationships). The figure specifically depicts homologous relationships among *PeqSPL* genes. Inter-species synteny analysis among *P. equestris*, *A. thaliana*, and *O. sativa* revealed that the *PeqSPL* genes exhibited limited syntenic relationships with their homologs in *A. thaliana* and *O. sativa* (Figure 5). There are four collinear gene pairs between *P. equestris* and *O. sativa* and just one pair identified between *A. thaliana* and *P. equestris*. Therefore, the closer genetic affinity between *PeqSPLs* and *OsSPLs* indicates a higher degree of conservation in monocotyledons.

### 2.6. Cis-Element Analysis Reveals Regulatory Potential of PeqSPL Promoters

**Figure 6:**
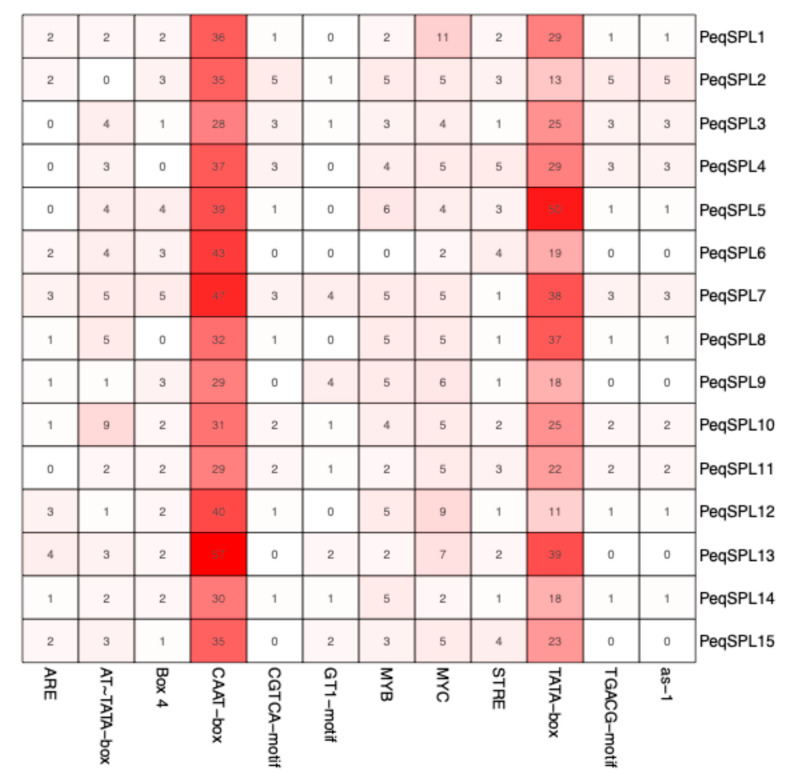
The number of *cis*-acting elements in the *PeqSPLs* promoter region. The top 10 most abundant *cis*-acting elements were listed. The red color represents the number of components, with deeper red indicating a larger number of components.

Multiple *cis*-acting elements were identified within the 2000 bp promoter regions upstream of the transcription start sites of *PeqSPL* genes (Figure S3; Table S3). A total of 72 types of *cis*-elements were obtained (Table S4), and the promoters of *PeqSPL5*, *PeqSPL7*, and *PeqSPL13* contain the most *cis*-elements (Table S4). The core promoter elements—CAAT and TATA boxes—were the most abundant (Figure 6). They are typically associated with transcriptional initiation or enhancer functions. Additionally, numerous elements associated with light responsiveness, phytohormone signaling, and anaerobic induction were detected. Among phytohormone-related elements, those responsive to jasmonic acid (JA) were predominant, followed by abscisic acid (ABA), auxin, and salicylic acid (SA). Elements linked to drought induction were also identified alongside anaerobic response motifs. These findings indicate that *PeqSPL* genes are likely regulated by diverse environmental and phytohormonal cues, supporting their multifunctional roles in developmental processes and stress adaptation.

### 2.7. Tissue-Specific Expression Patterns of PeqSPLs

**Figure 7:**
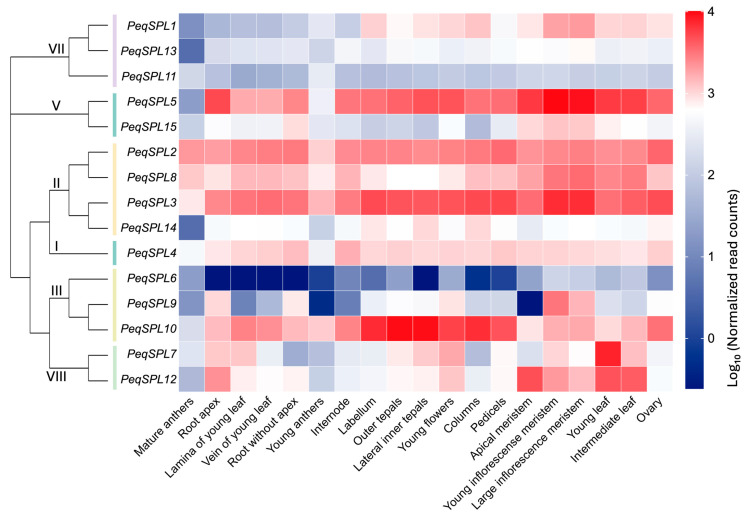
Heatmap of *PeqSPLs* expression patterns in different tissues based on online transcriptome data. Lines of different colors on the left represent different *SPL* subfamilies.

We investigated the tissue-specific expression patterns using RNA-Seq data from the online database (Table S5). RNA-Seq-based expression profiles showed that *PeqSPL* genes were differentially expressed in 19 tissues, including young inflorescence meristem, large inflorescence meristem, young flowers, outer tepals, lateral inner tepals, labellum, mature anthers, young anthers, columns, ovary, pedicels, young leaf, lamina of young leaf, vein of young leaf, intermediate leaf, root apex, root without apex, apical meristem, and internode (Figure 7). *PeqSPL* genes within the same subfamily (VII or II) exhibit high expression similarity. *PeqSPL4*, which exclusively belongs to subfamily I, demonstrates an expression pattern closely resembling that of the adjacent subfamily II. Genes in subfamily VII collectively display low expression levels, whereas those in subfamily II show uniformly high expression. In *P. equestris*, *SPL* genes of subfamily II likely play more critical functional roles compared to those in subfamily VII. *PeqSPL2*, *PeqSPL3*, *PeqSPL4*, *PeqSPL5*, *PeqSPL8*, and *PeqSPL10* were highly expressed across most tissues, suggesting their involvement in maintaining normal growth and development in *P. equestris*. In anthers, most *PeqSPL* genes exhibit low expression, with only *PeqSPL2*, *PeqSPL3*, *PeqSPL8*, and *PeqSPL10* showing elevated expression. Notably, *PeqSPL10* expression is high exclusively in young anthers, while *PeqSPL2*, *PeqSPL3*, and *PeqSPL8* maintain high expression in both young and mature anthers, implicating their roles in anthers development and maturation.

**Figure 8:**
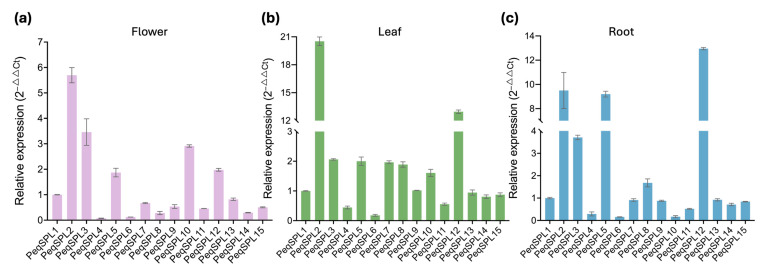
Expression analysis of *PeqSPL* genes in flower (**a**), leaf (**b**), and root (**c**) tissues based on qRT-PCR. Note: qRT-PCR data (2^−ΔΔCt^) for relative expression of *PeqSPLs*.

We further examined the expression levels of *PeqSPL* family genes in the flower, leaf, and root tissues of *P. equestris* using qRT-PCR. The reference gene used was *ACTIN* (LOC110036739), and the ΔCt of *PeqSPL1* in each tissue was used to calculate the 2^−ΔΔCt^ values for each gene. Consistent with the RNA-seq data (Figure 7), *PeqSPL2*, *PeqSPL3*, *PeqSPL5*, and *PeqSPL10* showed elevated expression in flowers; *PeqSPL2*, *PeqSPL3*, *PeqSPL5*, *PeqSPL7*, *PeqSPL8*, *PeqSPL10*, and *PeqSPL12* in leaves; and *PeqSPL2*, *PeqSPL3*, *PeqSPL5*, and *PeqSPL12* in roots of *P. equestris* (Figure 8). Among them, *PeqSPL2*, *PeqSPL3*, *PeqSPL5*, and *PeqSPL12* were highly expressed across most tissues, whereas *PeqSPL4*, *PeqSPL6*, and *PeqSPL11* remained at low levels, indicating that *PeqSPL2*, *PeqSPL3*, *PeqSPL5*, and *PeqSPL12* may play pivotal roles in sustaining normal growth and development of *P. equestris*.

## 3. Discussion

In this study, we identified 15 *SPL* genes in *P. equestris*, which were similar to the number of *SPLs* in other orchids, such as *Cymbidium goeringii* (16), *Dendrobium chrysotoxum* (17), *Gastrodia elata* (10), and *Dendrobium catenatum* (12) [25,34]. Phylogenetic analysis using SPL proteins from *A. thaliana* and *O. sativa* resolved eight SPL subfamilies. *PeqSPLs* were distributed in six subfamilies, with no members in subfamilies IV or VI. Similarly, *CgoSPLs*, *DchSPLs*, *GelSPLs*, and *DcaSPLs* lacked subfamily IV representatives, while subfamily VI contained three orchid *SPLs*: *DchSPL2*, *CgoSPL16*, and *DcaSPL3*. Notably, *G. elata* and *P. equestris* entirely lacked subfamily VI *SPLs*. Further analysis revealed that other monocots (*Chenopodium quinoa*, *Hordeum vulgare*) also lacked subfamily IV *SPLs* but retained subfamily VI members (*CqSPL18*, *HvSPL2*) [9,35]. In contrast, eudicots (*P. tomentosa*, *S. lycopersicum*, *M. domestica*, etc.) consistently harbored subfamily IV SPLs. These findings raise the hypothesis that subfamily IV SPLs (orthologs of AtSPL6) may have been lost during monocot evolution. *AtSPL6* plays a defensive role in Arabidopsis against pathogen infection, where pathogen-activated nuclear-localized TIR-NB-LRR receptors interact with AtSPL6 to positively regulate a subset of defense genes [36]. The absence of *AtSPL6* orthologs in monocots may compromise their immune defense mechanisms. It is also plausible that this loss could be essential for orchid species to establish mutualistic symbiosis with beneficial microorganisms. Among orchids retaining subfamily VI members, most possess only a single *SPL* gene in this subfamily [25,34]. This indicates that members of subfamily VI are functionally redundant and evolutionarily dispensable in orchid species. On the contrary, subfamily II represents the largest subfamily within the *PeqSPLs* family. Similarly, in other orchids and across monocots, subfamily II consistently harbors one of the highest numbers of SPL members [9,25,34,35]. This indicates that, in contrast to subfamilies IV and VI, subfamily II has undergone significant expansion in monocot lineages. Analysis of the *G. biloba* SPL gene family reveals divergence from *P. equestris*, with subfamily II containing only two members and subfamily III being the largest [3]. Comparative genomics showed minimal collinear conservation: only two collinear SPL pairs with *A. thaliana* and none with *P. tomentosa*.

Analysis of conserved protein motifs reveals that subfamily II members of PeqSPL possess not only the longest amino acid sequences among SPLs but also the highest number of conserved motifs (Table 1, Figure 2). Previous studies have identified a conserved C-terminal region exceeding 600 amino acids in subfamily II members of plant SPL [1]. And an Ankyrin repeat motif (IPR002110) is located approximately 500 amino acids downstream of the SBP domain, suggesting these domains may facilitate protein–protein interactions to modulate transcriptional regulation [7]. TMD predictions further indicate that all subfamily II members contain a C-TMD (Table S2). As a transcription factor within subfamily II, AtSPL14 not only regulates plant sensitivity to fumonisin B1 but also governs normal plant architecture [10]. Its homologous AtSPL16 is a nonfunctional duplicate, and its SBP domain may be lost due to frameshift mutations. In addition, *AtSPL1* and *AtSPL12* in subfamily II were widely expressed in almost all tissues examined, and exhibit conserved structural features characteristic of subfamily II members: SBP-box with nuclear localization signal sequence, Ankyrin domain for protein–protein interactions, and TMD [7]. Transient expression assays in tobacco leaves revealed weak cytoplasmic localization signals for AtSPL1 and AtSPL12. Critically, truncation of their TMDs abolished cytoplasmic localization, demonstrating that subcellular targeting to the cytoplasm is TMD-dependent. Subfamily II PeqSPL members also possess TMDs, and they may likewise exhibit dual localization in *P. equestris* cells to perform functions like those of AtSPL1 and AtSPL12. Although AtSPL1 and AtSPL12 act redundantly in conferring inflorescence thermotolerance via PYL-mediated ABA signaling during Arabidopsis reproductive development, their transcript levels remained uninduced by heat stress. This implies that their involvement in heat adaptation likely occurs through post-translational mechanisms (e.g., protein interactions or activation) rather than transcriptional upregulation. Subfamily II members exhibited expression widely across multiple tissues in *P. equestris* and might function in a basal mechanism that protects floral organs from environmental stresses. This highlights the evolutionary conservation and functional diversification of SPL transcription factors across plant lineages.

In Arabidopsis, subfamily I member AtSPL7 was considered to lack the TMD and ankyrin repeat domain found in AtSPL1 and AtSPL12 and was primarily involved in coordinating responses to light and copper [7,37]. In contrast, in *P. equestris*, PeqSPL4—also classified within subfamily I—possesses a TMD (Table S2). However, its overall protein sequence is shorter than that of subfamily II members and contains fewer conserved motifs. Therefore, whether PeqSPL4 shares functional similarity with subfamily II members in *P. equestris* remains to be further investigated.

*PeqSPL2, PeqSPL3*, and *PeqSPL5* exhibited broad expression with elevated levels across multiple tissues (Figure 7). *PeqSPL10* showed high expression in flower-related tissues, suggesting that they may play important roles in the flowering process of *P. equestris*. In anthers, only *PeqSPL2*, *PeqSPL3*, *PeqSPL8*, and *PeqSPL10* showed high expression. Notably, *PeqSPL10* expression underwent a marked reduction upon anther maturation. By manipulating these genes, it may be possible to regulate the timing of floral transition, increase the number of flowers per inflorescence, or alter the arrangement of flowers. Additionally, *PeqSPL2*, *PeqSPL3*, *PeqSPL5*, *PeqSPL8*, and *PeqSPL12* were highly expressed in meristems and leaves, indicating their potential involvement in leaf development and tissue formation. This tissue-specific expression divergence reflects functional diversification within the *PheSPL* gene family. In other orchids, *CgoSPL* showed minimal expression change, while *DchSPL9* and *GelSPL2* were significantly expressed during flowering [25]. In addition, *PtSPL* genes exhibited tissue-specific expression patterns, suggesting functional diversification across different plant tissues [5]. *GbSPL* members function as either activators or repressors depending on development or environment [3].

Our findings imply that PheSPL subfamily II members exhibit functional dominance through maximal motif complexity and TMD, enabling dual nuclear/cytoplasmic roles. Crucially, monocot-specific loss of subfamilies IV might suggest an evolutionary trade-off favoring symbiotic adaptation in orchids. These insights redefine SPL networks as key integrators of development and stress response in plants.

## 4. Materials and Methods

### 4.1. Whole-Genome Identification of SPL Gene in P. equestris

Using 16 AtSPLs and 19 OsSPLs protein sequences as reference, construct a Hidden Markov Model (HMM) with HMMER 3.0 (http://hmmer.janelia.org, accessed on 1 May 2025) based on these known SPL protein sequences. Subsequently, use this model to search all coding protein sequences of *P. equestris* to identify potential SPL family members. In parallel, perform BLASTP (ncbi-blast-v2.10.1+, http://blast.ncbi.nlm.nih.gov/Blast.cgi, accessed on 1 May 2025) alignments of all *P. equestris* protein sequences against the SPL reference sequences, with an E-value threshold of 1e-5. Sequences with significant alignments are retained as potential SPL candidates [38]. Merge all candidate sequences identified through the HMMER and BLASTP approaches to generate a preliminary set of candidate SPL protein sequences. Subject these candidates to domain annotation using pfamscan (v1.6, https://github.com/aziele/pfam_scan, accessed on 2 May 2025) and the Pfam A database (v33.1, ftp://ftp.ebi.ac.uk/pub/databases/Pfam/releases/, accessed on 2 May 2025), retaining only sequences containing PF03110 (SBP domain) as the final SPL sequences, yielding 15 confirmed sequences [39,40].

### 4.2. Physicochemical Properties of the SPLs

ExPASy (http://web.expasy.org/protparam/, accessed on 4 May 2025), an online analysis software, was used to analyze the physical and chemical properties of the obtained PeqSPLs proteins, including amino acid sequence length, molecular weight (MW), isoelectric point (pI), instability index (II), aliphatic index (AI), and hydrophilic large average (GRAVY) of the proteins. Subcellular localization of PeqSPL family members were predicted by online software (https://wolfpsort.hgc.jp/, accessed on 8 May 2025) [41].

### 4.3. Phylogenetic Analysis

Using the identified SPL protein sequences from *P. equestris*, *A. thaliana*, and *O. sativa*, construct a Neighbor-Joining (NJ) tree. Use MAFFT (v7, https://mafft.cbrc.jp/alignment/server/, accessed on 10 May 2025) to perform multiple sequence alignment [42]. Then construct the NJ tree using MEGA software (MEGA10) with parameter setting model = p-distance, missing data treatment = Partial deletion, cutoff = 50%, bootstrap replicates = 1000. iTOL online software (v6, https://itol.embl.de/, accessed on 10 May 2025) was used to annotate the evolutionary tree [43].

### 4.4. Protein Domain and Gene Structure Analysis

MEME software (v5.0.5, http://meme-suite.org/, accessed on 14 May 2025) was used to analyze conserved motifs in the PeqSPLs family [44]. The number of motifs to predict was set to 15. The phylogenetic tree and conserved protein motifs map were integrated using TBtools software (v2.309) [45]. Subcellular localization predictions for the PeqSPLs family members were performed using the online tool WoLF PSORT (https://wolfpsort.hgc.jp/, accessed on 14 May 2025) [41]. The membrane protein potential of PeqSPLs family members was assessed using DeepTMHMM (v1.0.8, https://dtu.biolib.com/DeepTMHMM/, accessed on 16 May 2025)), a software based on deep learning models for predicting transmembrane helices [46]. Based on the gff file, the gene structure was analyzed by online software GSDS (http://gsds.gao-lab.org/, accessed on 16 May 2025) [47].

### 4.5. Collinearity and Location Analysis on Chromosome

Collinearity analysis was performed using MCScanX with default parameters (MATCH_SCORE: 50; MATCH_SIZE: 5; GAP_PENALTY: -1; OVERLAP_WINDOW: 5; E_VALUE: 1e-05; MAX_GAPS: 25) to identify segmental duplications and tandem duplications arising from gene duplication events [48]. In alignment results, tandem duplication is classified when two aligned sequences are physically adjacent on a chromosome, indicating direct duplication of one sequence from the other. Segmental duplication is identified when multiple aligned sequence pairs cluster across different chromosomes, signifying that one genomic block duplicated another, thereby establishing collinearity (conserved gene order and orientation).

### 4.6. Cis-Acting Regulatory Elements Analysis

A 2 kb upstream region of each gene was extracted as the promoter regulatory sequence. TF binding sites on these promoters were predicted using PlantCARE (http://bioinformatics.psb.ugent.be/webtools/plantcare/html/, accessed on 22 May 2025) [49]. In the physical maps of gene promoters, the positions of binding sites were annotated and visualized, with only the top 12 most abundant TF families being displayed.

### 4.7. Expression Analysis

To investigate the expression patterns of *PeqSPL* genes, we analyzed RNA-seq data from 19 distinct tissues (including leaves, roots, floral organs, and shoot apical meristem) sourced from the publicly available Transcriptome Variation Analysis database (TraVA; https://travadb.org/, accessed on 5 June 2025) [50]. Normalized read counts extracted from TraVA underwent base-10 logarithmic transformation (Log_10_) to standardize visualization and analysis. Data is provided in Supplementary Tables S5. qRT-PCR assays were employed to examine the expression patterns of *PeqSPL* genes in flower, leaf, and root. According to previous studies, *ACTIN* (LOC110036739) was used as the internal reference gene, and primer sequences used in this experiment are provided in Table S6 [51].

## 5. Conclusions

In this study, 15 *SPL* genes were systematically identified in *P. equestris*, showing conserved SBP domains but diverse gene structures, motif compositions, and physicochemical properties. Phylogenetic analysis revealed closer evolutionary relationships with monocot species. Subfamily II members displayed complex structures, high expression across multiple tissues, and potential involvement in developmental processes such as flowering and meristem activity. Notably, several PeqSPLs possess predicted TMDs, suggesting noncanonical functions beyond transcriptional regulation. The presence of numerous *cis*-acting elements related to light response, hormones, and stress signals further supports their multifunctional regulatory roles. Overall, this study provides a foundational framework for understanding *SPL* gene functions and offers insights for future molecular breeding and functional genomics in orchids.

## Figures and Tables

**Table 1:** Basic information of *PeqSPL* gene family members.

Name	Gene ID	CDS ^1^ (bp)	Amino Acid ^2^ (No.)	MW ^3^ (Da)	pI ^4^	II ^5^	AI ^6^	GRAVY ^7^	Homolog ^8^
PeqSPL1	LOC110018951	1098	365	40729.45	5.88	57.13	62.27	−0.592	AtSPL13 [26]
PeqSPL2	LOC110021553	3045	1014	112500.42	6.28	58.01	80.5	−0.345	OsSPL6 [27]
PeqSPL3	LOC110021701	3321	1106	121984.69	7.11	59.63	74.98	−0.438	OsSPL15 [28]
PeqSPL4	LOC110022067	2490	829	93005.98	5.8	48.94	84.09	−0.251	OsSPL9 [29]
PeqSPL5	LOC110023454	1491	496	54162.83	8.63	46.46	57.08	−0.661	OsSPL3, OsSPL12 [30]
PeqSPL6	LOC110024613	1191	396	43610.53	9.07	59.48	49.07	−0.645	OsSPL5, OsSPL10 [31]
PeqSPL7	LOC110026415	1050	349	38391.89	8.99	50.13	55.7	−0.658	OsSPL14, OsSPL17 [18]
PeqSPL8	LOC110027702	2964	987	110148.37	7.3	46.62	82.88	−0.319	OsSPL6 [27]
PeqSPL9	LOC110030741	1035	344	37084.14	8.89	55.37	57.73	−0.538	AtSPL8 [32]
PeqSPL10	LOC110030902	546	181	19991.32	9.28	66.96	48.73	−0.959	OsSPL8 [33]
PeqSPL11	LOC110031164	942	313	34984.28	8.49	51.34	66.61	−0.485	AtSPL13 [26]
PeqSPL12	LOC110033253	1023	340	36708.28	8.57	57.42	60.88	−0.554	OsSPL14, OsSPL17 [18]
PeqSPL13	LOC110033310	1233	410	45150.41	6.13	57.77	67.29	−0.496	AtSPL13 [26]
PeqSPL14	LOC110033499	3246	1081	119828.82	8.33	50.77	75.76	−0.389	OsSPL15 [28]
PeqSPL15	LOC110039299	1419	472	52527.42	8.82	48.64	59.34	−0.719	OsSPL3, OsSPL12 [30]

Note: ^1^ CDS length of *PeqSPL* genes; ^2^ amino acids number of PeqSPL proteins; ^3^ molecular weights; ^4^ theoretical isoelectric points; ^5^ instability indexes; ^6^ aliphatic indexes; ^7^ grand averages of hydrophobicity; ^8^ PeqSPLs closest homologs from *A. thaliana* or rice.

## Data Availability

The original contributions presented in this study are included in the article/Supplementary Material. Further inquiries can be directed to the corresponding authors.

## Supplementary Materials

The following supporting information can be downloaded at https://www.mdpi.com/article/10.3390/plants14193090/s1, Figure S1: Sequence logos of the 15 protein motifs; Figure S2: *SPL* genes collinearity map within *P. equestris*; Figure S3: The composition and abundance of the top 10 most prevalent *cis*-acting elements in *PeqSPL* promoters; Table S1: Amino acids of SPL members; Table S2: Subcellular localization and transmembrane domain prediction of PeqSPL members; Table S3: *cis*-elements in the promoter of *PeqSPLs*; Table S4: Statistics of *cis*-elements in the promoter of *PeqSPLs*; Table S5: Expression pattern of *PeqSPLs*; Table S6: Primers sequences used in the qRT-PCR experiment.
