# Supplementary material for: Genome-Wide Identification and Expression Analysis of the SPL Gene Family in Phalaenopsis equestris
Source: Plants (Basel). 2025 Oct 7;14(19):3090. doi: 10.3390/plants14193090 (PMC12526091; doi:10.3390/plants14193090)
Supplement: Supplementary file 1 [file plants-14-03090-s001.zip › PeqSPL Figure S.pdf]

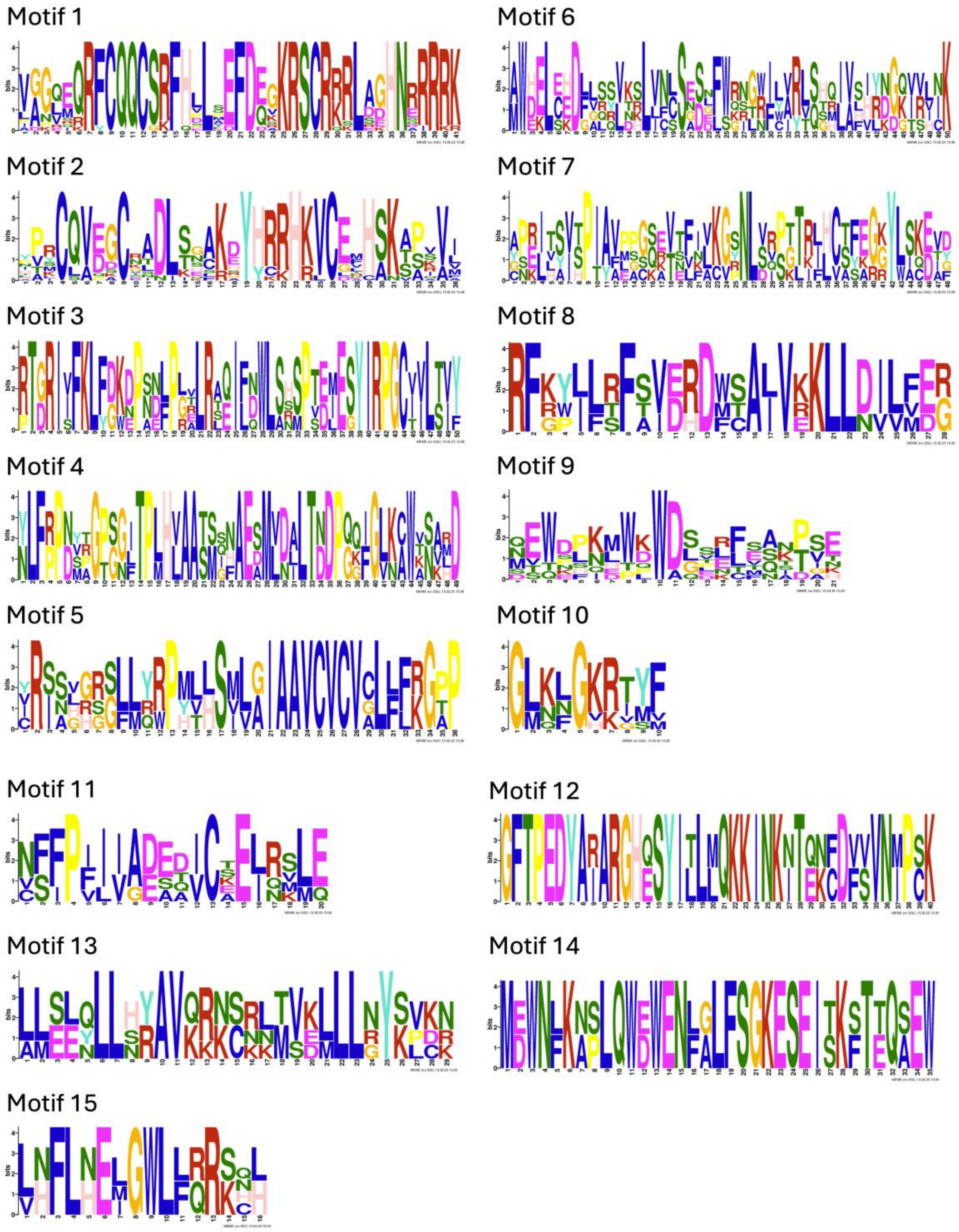

Figure S1. Sequence logos of the 15 protein motifs

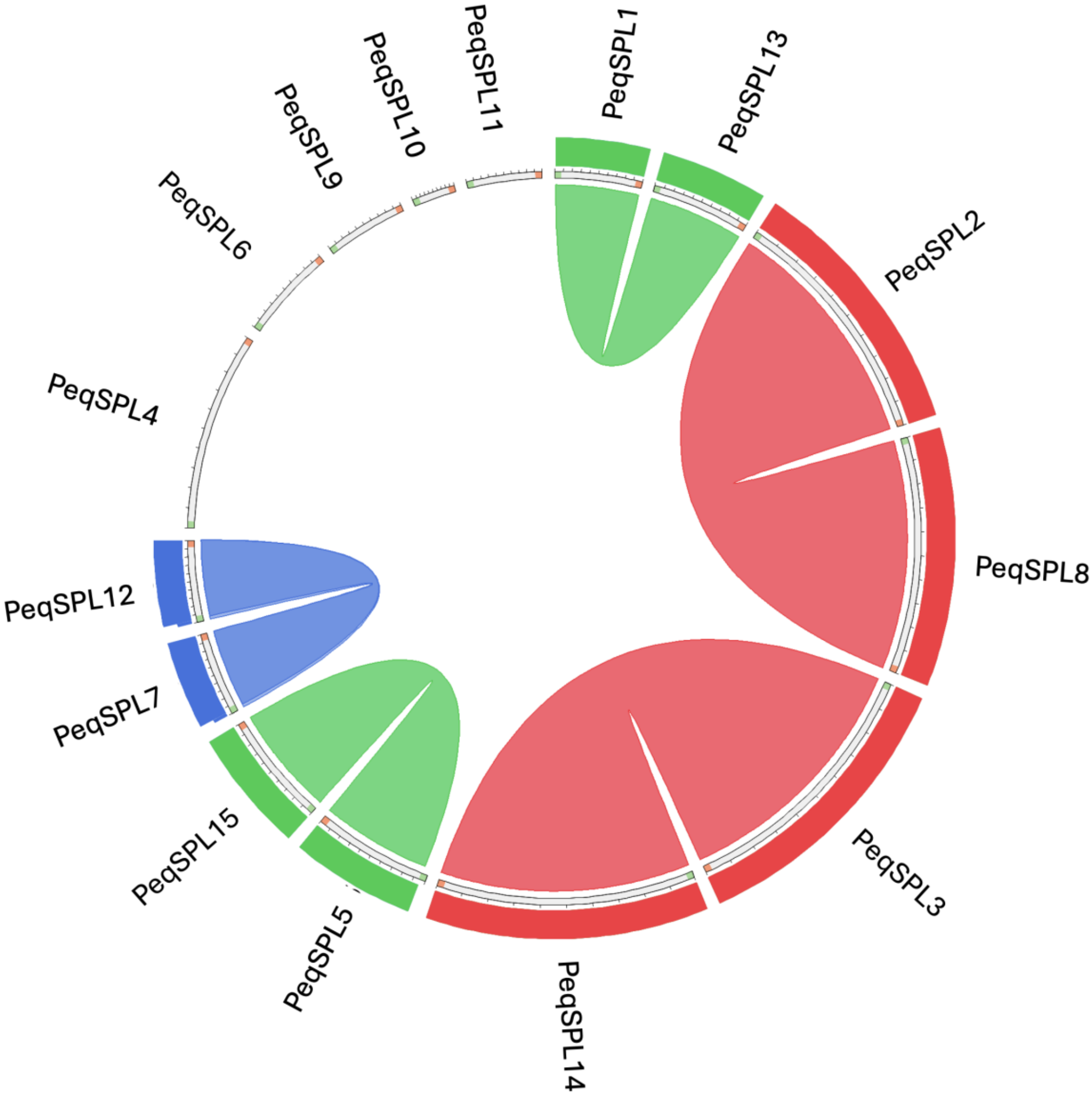

Figure S2. ***SPL*** **genes collinearity map within** ***P. equestris***

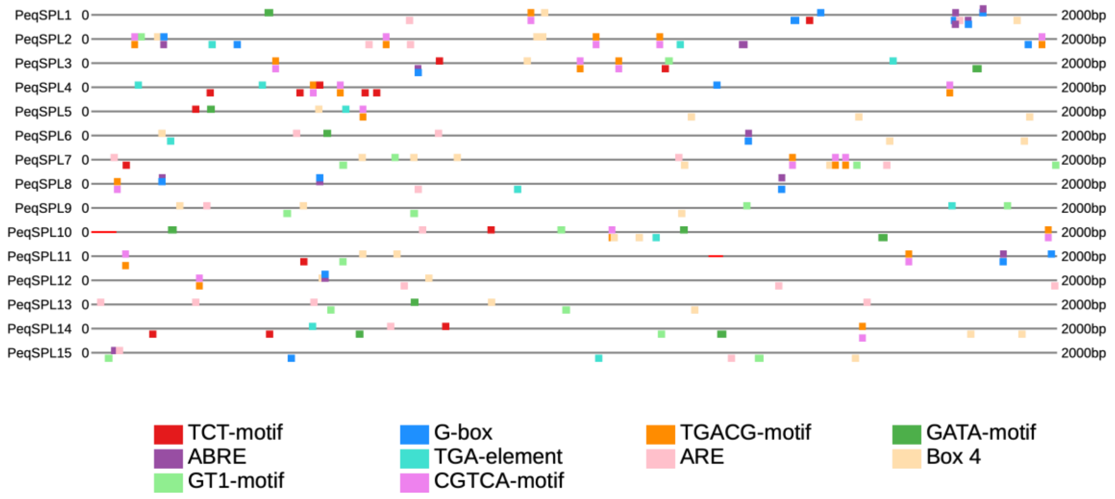

Figure S3. The composition and abundance of the top 10 most prevalent *cis*-acting elements in *PeqSPL* promoters. To present the distribution of these *cis*-elements more clearly, the core elements of CAAT-box and TATA-box were removed.
